# AAV-based gene therapy ameliorated CNS-specific GPI defect in mouse models

**DOI:** 10.1016/j.omtm.2023.101176

**Published:** 2023-12-14

**Authors:** Yoshiko Murakami, Saori Umeshita, Kae Imanishi, Yoshichika Yoshioka, Akinori Ninomiya, Takehiko Sunabori, Shibi Likhite, Masato Koike, Kathrin C. Meyer, Taroh Kinoshita

**Affiliations:** 1 Laboratory of Immunoglycobiology, Research Institute for Microbial Diseases, Osaka University, Suita, Osaka, Japan; 2 Graduate School of Frontier Bioscience, Osaka University, Suita, Osaka, Japan; 3 Center for Information and Neural Networks, National Institute of Information and Communications Technology (NICT), Osaka University, Suita, Osaka, Japan; 4 Center for Quantum Information and Quantum Biology, Osaka University, Suita, Osaka, Japan; 5 Central Instrumentation Laboratory, Research Institute for Microbial Diseases, Osaka University, Suita, Osaka, Japan; 6 Department of Cell Biology and Neuroscience, Juntendo University Graduate School of Medicine, Bunkyo-ku, Tokyo, Japan; 7 Center for Gene Therapy, Abigail Wexner Research Institute, Nationwide Children’s Hospital, Columbus, OH, USA; 8 Department of Pediatrics, The Ohio State University, Columbus, OH, USA; 9 Center for Infectious Disease Education and Research, Osaka University, Suita, Osaka, Japan

**Keywords:** inherited GPI deficiency, IGD, GPI-anchored protein, nestin-Cre, Pigafloxed, Rian locus, Rtl1

## Abstract

Thirty genes are involved in the biosynthesis and modification of glycosylphosphatidylinositol (GPI)-anchored proteins, and defects in these genes cause inherited GPI deficiency (IGD). *PIGA* is X-linked and involved in the first step of GPI biosynthesis, and only males are affected by variations in this gene. The main symptoms of IGD are neurological abnormalities, such as developmental delay and seizures. There is no effective treatment at present. We crossed *Nestin*-*Cre* mice with *Piga*-floxed mice to generate CNS-specific *Piga* knockout (KO) mice. Hemizygous KO male mice died by P10 with severely defective growth. Heterozygous *Piga* KO female mice are mosaic for *Piga* expression and showed severe defects in growth and myelination and died by P25. Using these mouse models, we evaluated the effect of gene replacement therapy with adeno-associated virus (AAV). It expressed efficacy within 6 days, and the survival of male mice was extended to up to 3 weeks, whereas 40% of female mice survived for approximately 1 year and the growth defect was improved. However, liver cancer developed in all three treated female mice at 1 year of age, which was probably caused by the AAV vector bearing a strong CAG promoter.

## Graphical abstract

**Figure:**
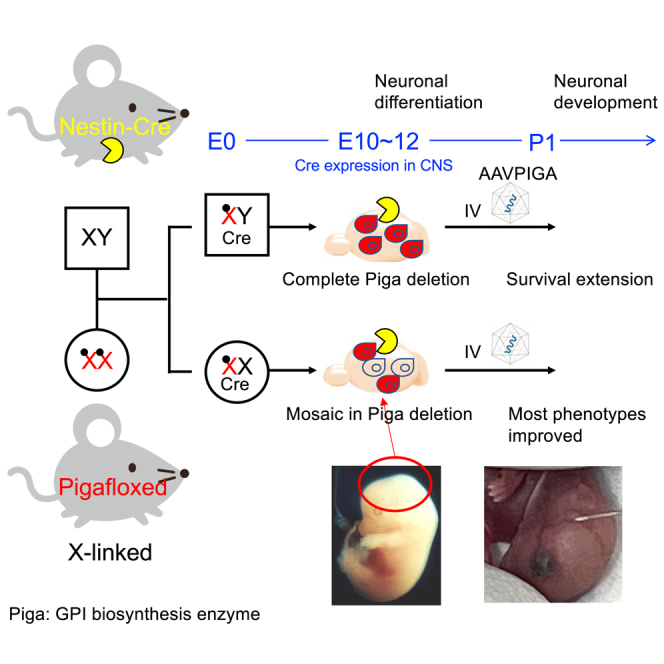

## Teaser

Neurological abnormalities due to defective expression of various GPI-anchored proteins in CNS caused by the Nestin-Cre-mediated *Piga* deletion during mouse embryogenesis is ameliorated by intravenous administration of scAAVPIGA immediately after birth. This suggests that neurological symptoms in patients with inherited GPI deficiency are reversible if treated early after birth.

## Introduction

Glycosylphosphatidylinositol (GPI) anchors various proteins to the plasma membrane. There are over 150 types of GPI-anchored proteins (GPI-APs) in mammalian cells, and they play various roles in fertilization, development, and immune responses as enzymes, adhesion molecules, receptors, and complement regulatory proteins.^1^ Thirty genes are involved in the biosynthesis and modification of GPI-APs, and variations in these genes can cause inherited GPI deficiency (IGD). Complete deficiency of GPI is lethal because the absence of over 150 GPI-APs on the cell surface is not viable; therefore, most IGD patients have a partial GPI deficiency. No effective treatment for IGD is available at present. To elucidate IGD pathology and to develop treatments, we and others previously generated IGD model mice, in which one of the genes required for GPI biosynthesis, phosphatidylinositol (PI) glycan anchor biosynthesis class O (*PIGO*) and *PIGV* gene, respectively, was partially deficient.^2^^,^^3^ We reported the effectiveness of adeno-associated virus (AAV)–based gene therapy in *Pigo*-deficient model mice using a gene editing strategy, homology independent targeted integration assisted with a low level of transgene expression.^2^

Whereas PIGO and PIGV function in the middle of the GPI biosynthesis pathway, PIGA is involved in the first step of GPI biosynthesis, the transfer of *N*-acetylglucosamine (GlcNAc) from UDP-GlcNAc to PI to generate GlcNAcPI. This step is mediated by the GlcNAc transferase complex consisting of PIGA, PIGH, PIGC, PIGP, PIGQ, PIGY, and DPM2.^1^ PIGA is a catalytic component and is essential for this reaction. *PIGA* is X-linked; therefore, only males who receive a variant allele from their mothers are affected. The main symptoms of *PIGA* deficiency are neurological abnormalities, such as developmental delay, intellectual disability, and seizures. To further investigate the effectiveness of AAV-based gene therapy of IGD, we generated CNS-specific *Piga* knockout (KO) mice by crossing CNS-specific Cre recombinase expressing *Nestin-Cre* mice with *Piga*-floxed mice.^4^ Nestin is expressed at approximately embryonic day (E) 7.5 during neuronal development.^5^ In the hemizygous *Piga* KO male mice, GPI-APs would be lost from neurons, astrocytes, and oligodendrocytes if Cre-mediated recombination occurs, whereas in the heterozygous *Piga* KO female mice, GPI-APs would be lost from half of those CNS cells, in which Cre-mediated recombination occurs because of X-inactivation. Similar to the previous report,^6^ male *Piga* KO mice die by approximately postnatal day (P) 10 after birth and have severely decreased levels of GPI-APs in the brain, whereas female *Piga* KO mice have a severe defect in myelination and die by approximately P25. Using these model mice as an evaluation system, we developed AAV-based gene therapy for *PIGA* deficiency. Here, we show that AAV-based gene replacement therapy is effective for improving some of the phenotypes of CNS-specific *Piga* KO mice. However, liver cancer developed in all three treated mice after 1 year. Although the occurrence of liver cancer has not been reported for AAV-based gene therapy in humans, careful consideration is needed in the dose, route of administration, and selection of suitable promoters for AAV-based gene therapy of IGD.

## Results

### Generation and phenotypes of CNS-specific *Piga* KO mice

**Figure 1:**
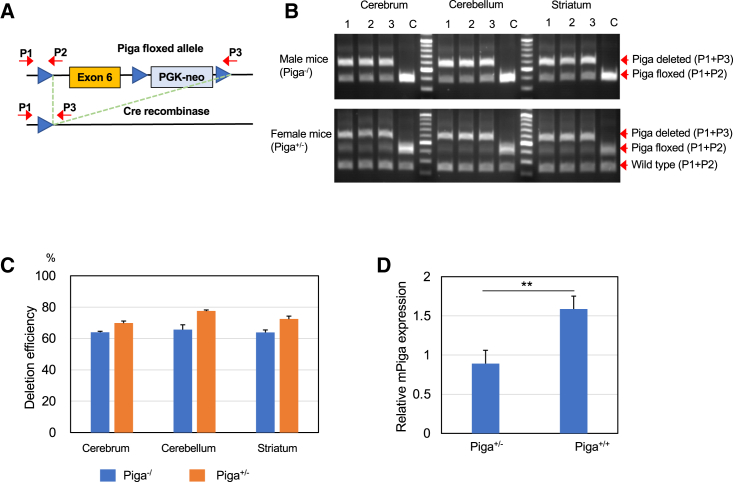
Deletion of *Piga* exon 6 by Cre recombinase in the CNS-specific lineage (A) Genotyping strategy to detect the *Piga*-floxed allele and the disrupted allele. See Materials and methods for details. (B) Genotypes of various parts of the brain from 3 *Piga*^*−/*^ and *Piga*^*+/−*^ mice at 5 days old compared with the wild type using competitive PCR analysis with P1, P2, and P3 primers (n = 3, each). Lane C, Genotypes of *Piga*-floxed mice as a negative control. (C) Average percentage of disrupted allele with respect to the *Piga*-floxed allele calculated from the band intensities of (B). Data are presented as mean ± SD. (D) Relative expression of *Piga* in the brains of *Piga*^*+/−*^ mice and wild-type littermates at 2 weeks of age (n = 4, each). Data are presented as mean ± SD. ∗∗p = 0.0011 (t test).

*Nestin-Cre* transgenic male mice were crossed with homozygous *Piga*-floxed female mice to induce Cre-mediated deletion of *Piga* exon 6 in neurons (Figure 1A). Percentages of exon 6-depleted alleles relative to *Piga*-floxed and wild-type alleles were calculated from the band intensities of the PCR products generated using three primers (Figure 1A and 1B). In male mice, the percentage of deleted (*Piga*^−^) alleles in *Piga*-floxed alleles was approximately 65% in various brain regions (hereafter, these mice are called *Piga*^*−/*^), suggesting that 35% of *Piga* expression levels remained. In female mice, the percentage of depleted alleles in *Piga*-floxed alleles was approximately 70% in various regions (hereafter, these mice are called *Piga*^*+/−*^) (Figure 1C). It has been reported that nestin is expressed not only in nerve cells but also in heart and skeletal muscle during embryogenesis.^7^^,^^8^ However, exon 6–depleted alleles were not detected in the heart and skeletal muscle of newborn *Piga*^*−/*^ and *Piga*^*+/−*^ mice (Figure S1). Considering the random inactivation of the X chromosome, *Piga* expression in the brains of *Piga*^+/−^ mice can be expected to be 65% (50% + 15%) of that of wild-type mice. qRT-PCR analysis revealed that the average *Piga* expression in the brains of *Piga*^+/−^ mice was approximately 56% of that in wild-type mice being in good agreement with the expectation, indicating that normal CNS cells did not proliferate dominantly relative to *Piga*-deleted cells during development in *Piga*^+/−^ mice (Figure 1D).

**Figure 2:**
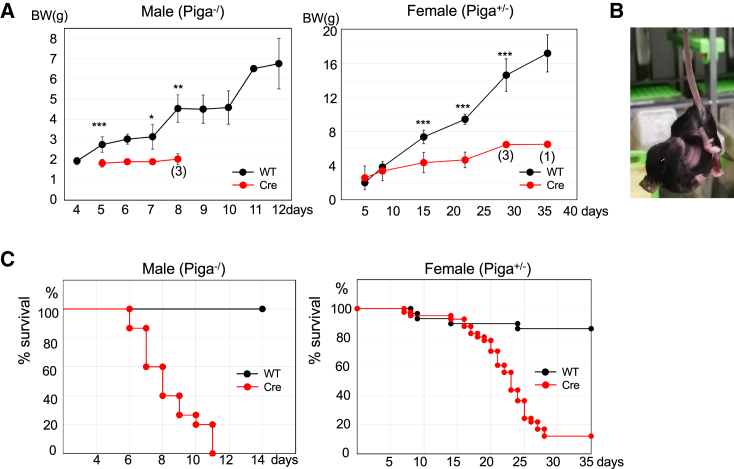
Phenotypes of *Piga*^*−/*^ and *Piga*^*+/−*^ mice (A) Growth curves of the mice. Data are presented as mean ±SD. *Piga*^*−/*^ mice (n = 14), wild-type males (n = 15), *Piga*^*+/−*^ mice (n = 41), wild-type females (n = 29). Numbers in parentheses toward the bottom of the graph indicate the number of mice alive. ∗p = 0.049; ∗∗p = 0.008; ∗∗∗p < 0.001 (t test). (B) *Piga*^*+/−*^ mice shown hindlimb clasping. (C) Survival curves. *Piga*^*−/*^ mice (n = 15), wild-type males (n = 15), *Piga*^*+/−*^ mice (n = 41), wild-type females (n = 29).

**Figure 3:**
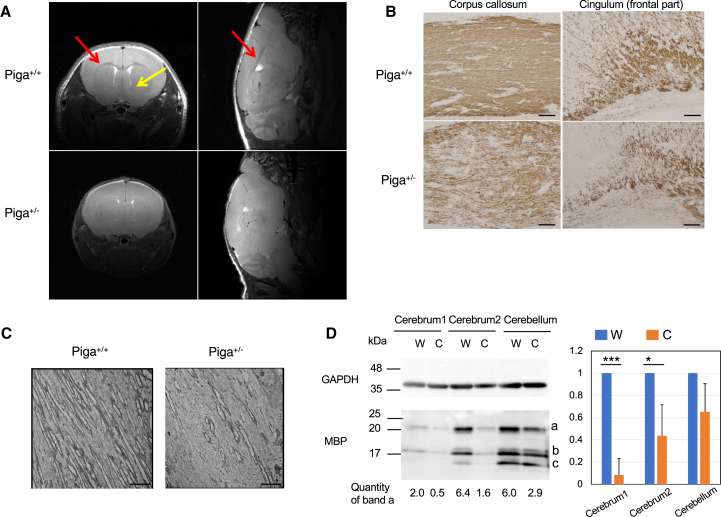
Defect in myelination in the brain of *Piga*^*+/−*^ mice at 21 days old (A) *In vivo* T_2_ weighted brain MRI of a *Piga*^*+/−*^ mouse compared with a wild-type littermate. Left, coronal section; right, sagittal section of the brain. Red arrows, corpus callosum; yellow arrow, anterior commissure. (B) Corpus callosum (left) and cingulum (right) stained with an anti-MBP antibody. (C) Nerve fibers of the corpus callosum observed by electron microscopy. (D) Left, western blot of brain tissue using an anti-MBP antibody. Cerebrum1, part of the cerebrum anterior to Bregma; Cerebrum2, part of the cerebrum posterior to Bregma; W, wild-type littermate; C, *Piga*^*+/−*^. These are the representative data of the 3 repeated experiments. a, b, and c are the splicing variants of MBP. MBP band a intensities were normalized with those of GAPDH, loading controls. Right, relative expression of MBP (band a) in *Piga*^*+/−*^ mice compared with wild-type littermates (n = 3). ∗p = 0.025; ∗∗∗p < 0.001(t test). Scale bar: 100 μm in (B), 500 nm in (C).

*Piga*^+/−^ and *Piga*^−/^ mice showed severely defective growth. *Piga*^+/−^ mice started limb clasping and ataxic gait from approximately P10, and these symptoms were progressive (Figures 2A and B). Survival time was drastically shortened; the *Piga*^+/−^ mice died by approximately P25 and the *Piga*^−/^ mice died by approximately P10 (Figure 2C). Brain MRI of *Piga*^+/−^ mice at P21 showed defective myelination; while myelination progressed in the corpus callosum and anterior commissure of wild-type brains, as indicated by the appearance of low-intensity regions (Figure 3A, red and yellow arrows, respectively), corresponding regions in *Piga*^+/−^ brain maintained high intensities (Figure 3A). Myelination defect in *Piga*^+/−^ mice was confirmed by (1) sparse staining of the major myelin protein, myelin basic protein (MBP), in the corpus callosum and cingulum compared to wild-type brains (Figure 3B), (2) decreased numbers of myelinated nerve fibers bearing electron-dense myelin sheath (Figure 3C), and (3) decreased levels of MBP in *Piga*^+/−^ cerebrum and cerebellum (C) compared to wild-type tissues (W) as determined by western blotting (Figure 3D).

### AAV-PHPeB-mediated expression of human *PIGA (hPIGA)* cDNA prolonged survival time of CNS-specific *Piga* KO mice

**Figure 4:**
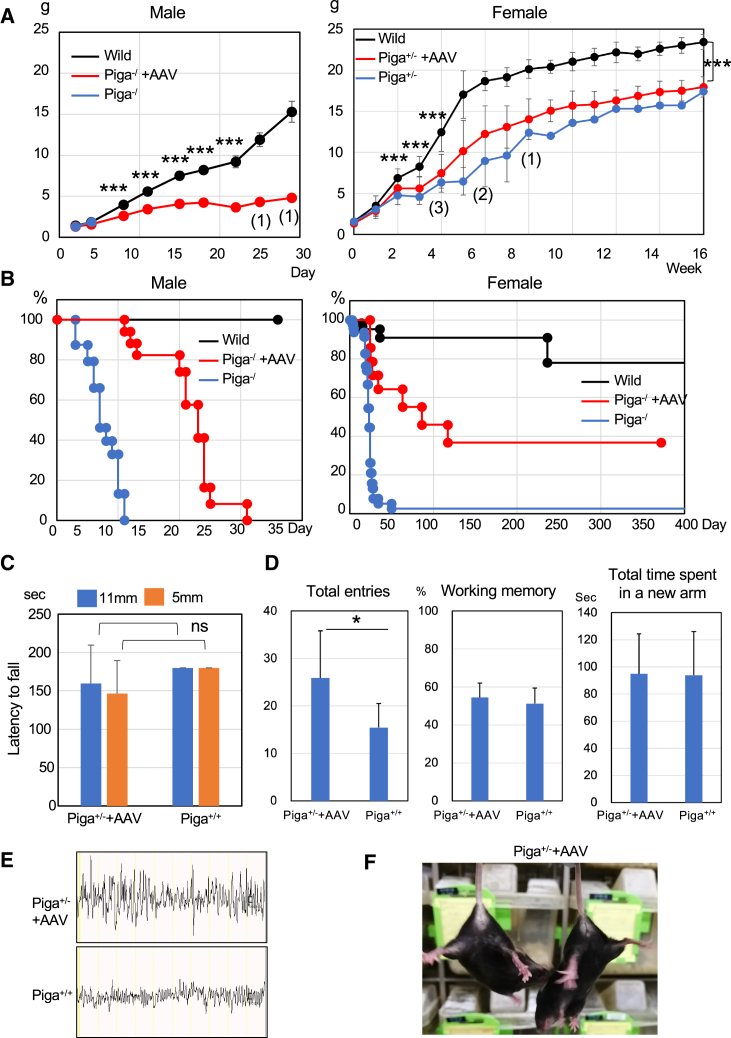
Effect of AAV treatment on *Piga*^*−/*^ and *Piga*^*+/−*^ mice (A) Growth curves. Vertical axis, body weight: horizontal axis, days or weeks after birth. Numbers in parentheses toward the bottom of the graph indicate the number of mice alive. Data are presented as mean ± SD. *Piga*^*−/*^ mice (n = 11), AAV-treated *Piga*^*−/*^ mice (n = 16), wild-type males (n = 21), *Piga*^*+/−*^ mice (n = 41), AAV-treated *Piga*^*+/−*^ mice (n = 14), wild-type females (n = 19). ∗∗∗p < 0.001 (wild versus AAV-treated mice, t test). (B) Survival curves. Vertical axis, percentage of survival: horizontal axis, days after birth. *Piga*^*−/*^ mice (n = 24), AAV-treated *Piga*^*−/*^ mice (n = 20), wild-type males (n = 51), *Piga*^*+/−*^ mice (n = 47), AAV-treated *Piga*^*+/−*^ mice (n = 17), wild-type females (n = 63). (C) Hanging test at 60 days old using 11- and 5-mm wire mesh (n = 7 each). Data are presented as mean ± SD. (D) Y-maze test. Data are presented as mean ± SD (n = 10 each, ∗p = 0.02, t test). (E) Representative EEG recordings in AAV-treated *Piga*^*+/−*^ mice showing the background activity in AAV-treated *Piga*^*+/−*^ mice and their wild-type controls. (F) The AAV-treated *Piga*^*+/−*^ mouse did not show hindlimb clasping.

*Piga*^*+/−*^ and *Piga*^−/^ mice were intravenously administered 10^11^ virus genomes (vg)/mouse of AAV-PHPeB *CAG*-*HA-hPIGA* (AAV-*hPIGA*) at P1 or P2. The growth defect was only partially restored in *Piga*^*+/−*^ and *Piga*^−/^ mice (Figure 4A), but survival time was significantly extended for both genotypes. Of the *Piga*^+/−^ mice, 37% survived until approximately 1 year of age and 50% of *Piga*^*-*/^ mice survived until P21 (Figure 4B), indicating that self-complementary (sc) AAV treatment was effective as early as 1 week after administration because most of the nontreated *Piga*^*−*/^ mice died by P10. The time course analysis of AAV-derived *hPIGA* expression in the wild-type mice revealed that it was expressed at the level almost similar to the endogenous level at day 4 after injection of AAV (Figure S2).

### Neurological amelioration of AAV-*hPIGA*-treated mice

In the hanging test, AAV-*hPIGA*-treated *Piga*^+/−^ mice showed an ability comparable to that of wild-type mice (Figure 4C). The Y-maze test measured total entries, special working memory, and time spent in the new arm (Figure 4D). Of these, AAV-*hPIGA*-treated *Piga*^+/−^ mice showed a significantly higher value only for the total number of entries, and the other two did not differ from controls, suggesting that AAV-*hPIGA*-treated *Piga*^+/−^ mice were restless and hyperactive (Figure 4D, left). Electroencephalogram (EEG) analysis of AAV-*hPIGA*-treated *Piga*^+/−^ mice showed that high voltage with slightly slower waves was prominent in the dark phase (Figures 4E and S3). They showed no spontaneous seizures, but tonic-clonic seizures (score 4, Table S1) were induced in one out of four mice that received a low dose of pentylenetetrazole (20 mg/kg), indicating mild susceptibility to seizures (Figure S3). We could not test the untreated *Piga*^+/−^ mice for comparison in these neurological tests because their neurological abnormalities were too severe, and they do not survive past P25.

Myelination of AAV-*hPIGA*-treated *Piga*^+/−^ mice was delayed at P19, as in nontreated mice (Figure S4A). Myelination of AAV-*hPIGA*-treated *Piga*^+/−^ mice had progressed at P54 but to a lesser extent than in wild-type mice (Figure S4B).

Brain MRI showed that in AAV-*hPIGA*-treated *Piga*^−/^ mice at P17, the length of the corpus callosum was shorter and the cerebellum was smaller than in their wild-type littermates (Figures S5A and S5B), suggesting that the structural abnormalities of the brain could not be improved by the gene replacement after birth. To note, none of the untreated *Piga*^−/^ mice were available for comparison at P17 because they did not survive past P10.

**Figure 5:**
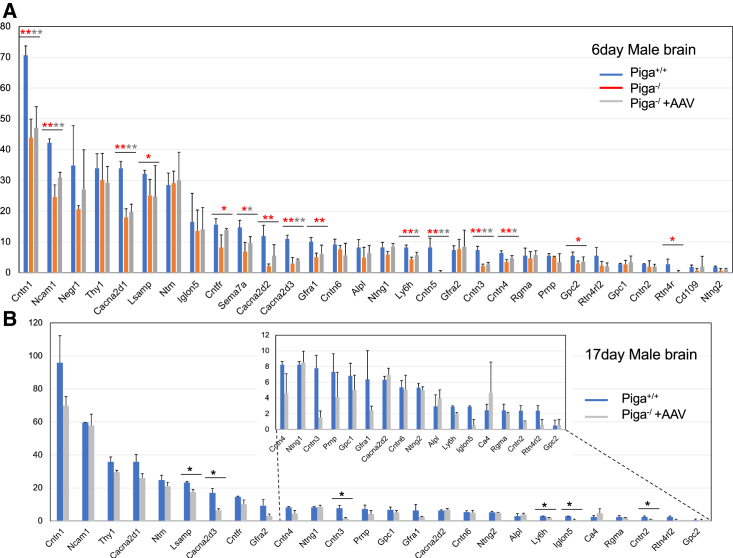
Proteomic analysis of AAV-treated or nontreated *Piga*^*−/*^ mice compared with wild-type littermates (A) AAV-treated or nontreated *Piga*^*−/*^ mice compared with wild-type mice at 6 days old (n = 3 each). (B) At 17 days old, averages of relative quantitative values (calculated by Scaffold 5) were compared. Data are presented as mean ± SD (n = 2 each). The vertical axis showed the relative quantitative values analyzed with Scaffold 5. ∗p < 0.05; ∗∗p < 0.01 (t test). Red color, wild-type mice versus *Piga*^*−/*^ mice; gray color, treated versus untreated *Piga*^*−/*^ mice.

Proteomic analysis of *Piga*^−/^ mouse brains revealed that Contactin 1–6 (CNTN1–6) levels, except for CNTN6, were severely decreased at P6 and not rescued by AAV-*hPIGA* administration at P6 or P17 (Figures 5A and B). CNTN1–6 are GPI-APs and occur in membrane-bound and soluble forms. CNTN1 is indispensable for paranodal junction formation. It also plays an important role in myelination. Therefore, decreased levels of CNTN1 may have contributed to the phenotype of *Piga*^+/−^ mice. As for other GPI-APs, levels of voltage-gated calcium channel alpha2/delta subunit 1–3 (CACNA2D1–3) were severely decreased in *Piga*^−/^ mice at P6 and were partially rescued by AAV-*hPIGA* treatment at P17. Levels of glial cell line–derived neurotrophic factor (GDNF) family receptor alpha 1,2 and Reticulon 4 receptor-like 2 were also decreased but not rescued by AAV-*hPIGA* treatment. Levels of immunoglobulin (Ig)-like cell adhesion (family members, such as NTRM, LSAMP, NEGR1, and IgLON5, and Netrin-G1 and G2 were not or were mildly decreased in *Piga*^−/^ mice at P6 and were partially rescued by AAV-*hPIGA* at P17 (Figures 5A and B).

### Evaluation of *hPIGA/Piga* levels in AAV-*hPIGA*-treated mice

**Figure 6:**
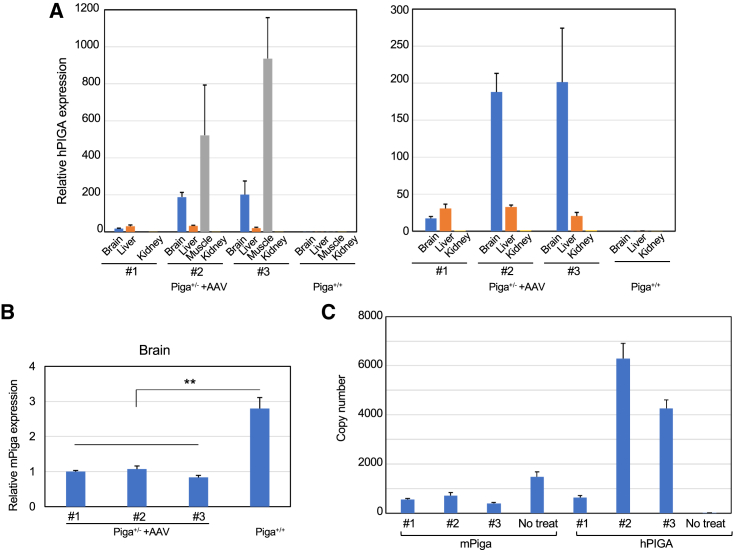
Relative expression of transgene *hPIGA* and endogenous *Piga* in AAV-treated *Piga*^*+/−*^ mice at 1 year old (A) Left, relative expression of transgene *hPIGA* in various tissues in 3 AAV-treated *Piga*^*+/−*^ mice and a wild-type littermate (muscle data from no. 1 mouse is missing); right, the same data as left but without muscle data. *hPIGA* expression in the kidney of the no. 1 mouse was set to 1. (B) Relative expression of endogenous *Piga* in the brain (cerebrum). *Piga* expression in the no. 1 mouse was set to 1. The data were from the triplicate analysis and are presented as mean ± SD. AAV-treated *Piga*^*+/−*^ mice (n = 3); wild-type females (n = 1). ∗∗p = 0.005 (t test). (C) Copy number of AAV-derived *hPIGA* and endogenous *mPiga* mRNA were compared in AAV-treated mice and a nontreated mouse.

AAV-PHPeB-derived *hPIGA* expression in various tissues from three *Piga*^+/−^ mice of approximately 1 year of age was analyzed. qRT-PCR analysis revealed that AAV-*hPIGA* treatment resulted in widespread and robust expression of *hPIGA* in the brains of treated *Piga*^+/−^ mice, whereas in the periphery, *hPIGA* expression was highest in skeletal muscle and was low but significant in the kidney (Figure 6A). Surprisingly, the expression of endogenous *Piga* was significantly decreased in the brains of AAV-*hPIGA*-treated *Piga*^+/−^ mice compared with the levels in nontreated *Piga*^+/−^ mice (33% in Figure 6B versus 56% in Figure 1D), suggesting potential endogenous feedback regulation of *Piga* expression. *hPIGA* was N-terminally tagged with hemagglutinin (HA) and anti-HA immunohistochemistry of brain tissues predominantly stained nerve cells (Figure S6). Copy numbers of AAV-derived *hPIGA* mRNA were compared with those of endogenous *Piga* mRNA in these mice. Two out of three mice showed *hPIGA* expression above the endogenous *mPiga* expression level even after 10 months (Figure 6C).

### Hepatocellular carcinoma (HCC) after AAV-PHPeB gene delivery in *Piga*^+/−^ mice

**Figure 7:**
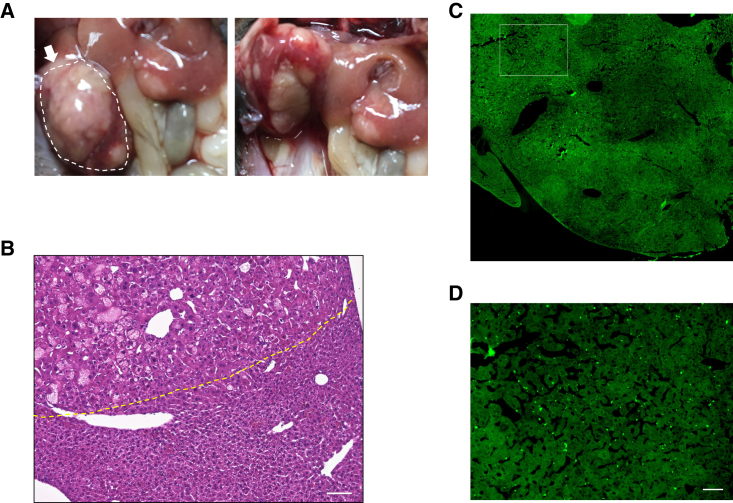
Liver cancers developed in-AAV treated aged *Piga*^*+/−*^ mice (A) Photographs from 2 directions—front and back of multiple liver tumors in a 1-year-old mouse (no. 3). (B) H&E staining of the tumor (60× magnification; scale bar, 100 μm), the part enclosed by white dotted line in (A). The upper left (above the yellow dotted line) is a typical HCC with conspicuous cell dysplasia. (C) A navigation image of the whole liver tumor in (B) stained with an anti-HA antibody followed by an FITC-conjugated secondary antibody. (D) Image of the squared area in (C), 11.1× magnification; dots are nonspecific staining (see Figure S5C with secondary antibody only). Scale bar, 100 μm.

**Figure 8:**
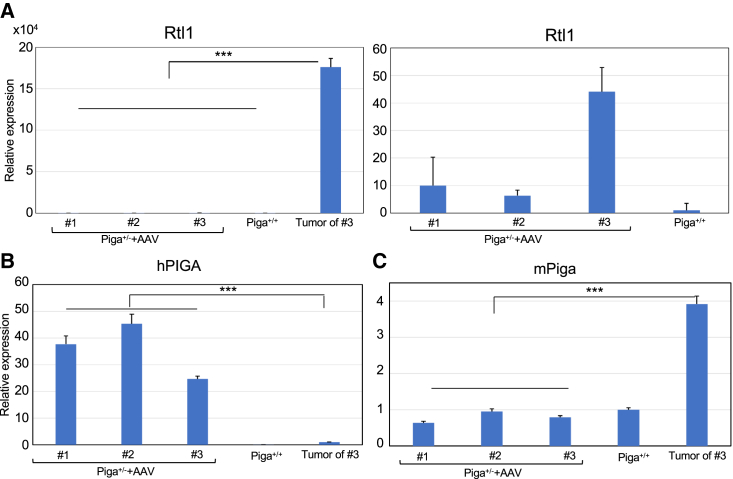
Relative expression of oncogene, *Rtl1*, transgene *hPIGA*, and endogenous *Piga* in the liver of AAV-treated aged *Piga*^*+/−*^ mice, which developed liver tumors (A) Relative expression of *Rtl1* in normal parts of the liver and in tumors of mice. The graph on the right shows data from normal liver only and the graph on the left includes the tumor data. *Rtl1*expression in a *Piga*^*+/+*^mouse was set to 1. Normal liver from AAV-treated *Piga*^*+/−*^ mice (n = 3); liver tumor (n = 1). (B) Relative expression of transgene *hPIGA* in normal liver and in tumors. *hPIGA* expression in the kidney of mouse no. 1 (Figure 6A) was set to 1. (C) Relative expression of endogenous *Piga* in normal liver and in tumors. *Piga* expression in a *Piga*^*+/+*^mouse was set to 1. The data were from the triplicate analysis and are presented as mean ±SD. (B and C) Normal liver from AAV-treated *Piga*^*+/−*^ mice (n = 3); normal liver from wild-type mouse (n = 1); liver tumor (n = 1). ∗∗∗p < 0.001 (t test).

Although AAV treatment has been largely reported as safe and well tolerated in rodents and larger animals and even in humans, there are reports that described the development of HCC in mice after the systemic delivery of AAV gene therapy vectors.^9^^,^^10^ In our study, liver tumors were found in all three *Piga*^+/−^ mice that were euthanized for gene expression analysis at approximately 1 year of age. Figure 7A shows representative tumor images of one mouse. It was proven to be pathologically cancerous by H&E staining (Figure 7B). Anti-HA staining showed that the *hPIGA* transgene was not overexpressed in the tumor (Figures 7C, 7D, and S7). Because HCC development was attributed to AAV integration into the RNA imprinted and accumulated in nucleus (*Rian*) locus and the resulting overexpression of proximal microRNAs and retrotransposon-like 1(*Rtl1*),^10^ liver tissues of normal appearance from three AAV treated *Piga*^+/−^ and one *Piga*^+/+^ mice and the liver tumor from one of the AAV treated *Piga*^+/−^ mice were analyzed by qRT-PCR for the expression of *Rtl1* and *hPIGA* (Figures 8A and 8B). Expression of *Rtl1* was drastically increased in the tumor (approximately 30,000 times that of wild-type liver) and other liver tissues (6–40 times that of wild-type liver), suggesting AAV integration into the *Rian* locus (Figure 8A). These findings were consistent with the liver tissues of pathological appearance in fact being cancerous. In contrast, the expression of *hPIGA* was decreased in the tumor tissue (Figure 8B), which is consistent with the result of the immunohistochemical staining for HA-hPIGA (Figures 7C, 7D, and S7). Unexpectedly, the expression of endogenous *Piga* was increased in the tumor (Figure 8C); however, the expression of *Pigo,* another GPI biosynthesis gene, or *Glypican3*, the highly expressed GPI-AP in the liver cancer, was not drastically increased in the tumor (Figure S8).

## Discussion

PIGA is a catalytic subunit of the enzyme complex involved in the first step of GPI biosynthesis. Because *PIGA* is an X-linked gene, males who receive the maternal pathogenic allele develop the disease. Therefore, the frequency of *PIGA* deficiencies is higher than that of other IGDs, with approximately 100 patients reported worldwide up to now.^11^^,^^12^^,^^13^ Human females who are heterozygous for pathogenic *PIGA* alleles are predicted to be mosaic for GPI-AP expression because of X-inactivation. These females develop normally and become healthy carriers of *PIGA* pathogenic alleles.^12^ Fluorescence-activated cell sorting analysis of granulocytes revealed that one carrier showed two peaks (normal and decreased) of CD16 expression, whereas others showed normal expression.^12^ X-inactivation occurs randomly early in development, but GPI^+^ cells become dominant, especially in tissues in which GPI-APs are critical during development, such as neuronal tissues. However, this does not seem to be true in mice. Human cytomegalovirus (CMV)-Cre mice crossed with *Piga*-floxed female mice generate hemizygous total-KO *Piga*^−/^ male and heterozygous total-KO *Piga*^+/−^ female mice, both of which are lethal. Total-KO *Piga*^−/^ male embryos die at E9 and Het-KO *Piga*^+/−^ female embryos die at E13, with severe malformation.^14^ We attempted several times to generate *Piga* knockin mice bearing a patient’s variant allele either by direct injection of Cas9 with gRNA and donor oligo into fertilized eggs or by standard ES cell methodology, but our attempts failed. We were unable to generate heterozygous females (data not shown). Instead, all of the reported *Piga* KO models have been based on CNS-specific KO mice.^6^^,^^15^

To determine whether a gene therapy approach is useful for *PIGA*-IGD, we treated CNS-specific KO mice with an AAV-based gene therapy. *Nestin-Cre* is active as early as E7.5; significant recombination is detected in the developing brain as well as in the neural tube at E10.5–11.5, and recombination in the CNS is almost complete by E15.5.^16^ In Nestin-Cre/*Piga*-floxed mice, neurons, astrocytes, and oligodendrocytes were defective in *Piga*. The *Piga* gene was deleted in approximately 70% of the brain DNA in the floxed allele of *Piga*^+/−^ females (Figure 1C), which indicated that *Piga* expression would be 65% of that in wild-type mice if random inactivation of X chromosome took place. Actually, endogenous *Piga* expression was approximately 56% of that in wild-type mice (Figure 1D), indicating that normal CNS cells did not proliferate dominantly against *Piga*-deleted cells in these mice. Although *Piga*^−/^ males and *Piga*^+/−^ females were born alive, they showed severely defective growth and defective myelination and most of them died by P10 and P25, respectively (Figures 2 and 3).

Intravenous injection of scAAV-PHPeB-*CAG*-*hPIGA* at P1–P2 was very effective at extending their survival; half of the *Piga*^−/^ males lived up to 3 weeks and 40% of the *Piga*^+/−^ females lived more than 1 year (Figure 4). The growth defect was not completely rescued, but significant improvements in neurological phenotypes, such as muscle weakness and limb clasping, were observed (Figure 4). Brain MRI showed improved myelination in AAV-hPIGA-treated *Piga*^+/−^ females at P54, and no spontaneous seizures were observed, indicating no prominent neurological abnormality.

Proteomic analysis of the brains of *Piga*^−/^ male mice revealed that levels of CNTN1–6 were severely decreased at day 6 and not rescued by AAV-*hPIGA* administration at P6 or P17 (Figures 5A and 5B). CNTN1 is indispensable for early interactions between axons and glia. CNTN1 clusters at the paranodal junction, establishing a complex with contactin-associated proteins (CNTNAPs, Casprs) and interacts with glial neurofascin-155 to establish axon glial contacts for the insulating function of myelin.^17^ CNTN1 is essential for the proper localization of potassium Kv1.2 channels at juxtaparanodal regions, indicating that it is needed for correct action potential repolarization during action potential conduction.^17^ The defect in nerve conduction and excitability of the target muscles may cause muscle atrophy, which is consistent with hypotonia in human patients and hindlimb weakness in *Piga*^−/^, *Piga*^+/−^, and *Cntn1* KO mice. *Cntn1* KO mice are severely growth restricted and have a myelination defect and cerebellar dysfunction and die at approximately 3 weeks of age;^18^ these phenotypes are similar to those of *Piga*^+/−^ mice. A human infant with a homozygous variation in the *CNTN1* gene has been reported. Their phenotype was lethal myopathy because of decreased levels of CNTN1 at neuromuscular junctions, leading to disrupted communication between muscles and nerves.^19^ CNTN1 also plays an important role in myelination. The maturation and differentiation of oligodendrocytes is controlled by CNTN1-dependent signal transduction through its interaction with PTPRZ, NOTCH, PTPα, and FYN on oligodendrocytes and their precursor cells.^20^ Therefore, the decreased expression of CNTN1 would contribute to the phenotype of *Piga*^−/^ and *Piga*^+/−^ mice and of human IGD cases. The levels of CACNA2D1–3 were also severely decreased at day 6 of *Piga*^−/^ mice and were partially rescued by AAV-*hPIGA* treatment at P17. CaVs express as a heteromeric proteins on the plasma membrane of skeletal muscles and neurons, in which the α1 subunit is associated with two auxiliary subunits, the intracellular β subunit, and the α2δ subunits; the latter are encoded by four genes, CACNA2D1–4, and are reported to be GPI-APs.^21^ They play important roles in the trafficking and function of the CaV channel complexes. There are several reports that the defect in CACNA2D1 or CACNA2D2 causes developmental and epileptic encephalopathies, hypotonia, and severe cerebellar ataxia,^22^ symptoms of which can be also observed in IGDs, including PIGA deficiencies.^12^ Therefore, the decreased expression of *CACNA2D1–3* (Figure 5) may contribute to the mouse phenotypes such as muscle weakness and ataxic gate. Other important GPI-APs include the GDNF receptor family of proteins, GFRα-1,2, which are involved in various signaling pathways for neuronal cell survival and migration through activation of RET tyrosine kinase receptor. *RET* and *GFRα-1* are the responsible genes for Hirschsprung disease,^23^ from which severe cases of IGD suffer.^24^ In the mouse models, we did not find any organ abnormalities. Tissue nonspecific alkaline phosphatase (TNAP), a GPI-AP, was mildly decreased at day 6 of *Piga*^−/^ mice. Decreased surface expression of TNAP is responsible for seizures in IGD cases. TNAP dephosphorylates pyridoxal phosphate (PLP) to PL, a membrane permeable form of vitamin B_6_, which is converted to PLP intracellularly and functions as a cofactor for GABA synthase. *TNAP* KO mice developed seizures due to decreased GABA levels in the brain, which was rescued by PL treatment.^25^ Likewise, the administration of pyridoxin is very effective in controlling seizures in some IGD cases.^24^ TNAP is also involved in the uptake of vitamins B_1_ and B_2_, the latter of which requires another GPI-AP, CD73, which also converts flavin adenine dinucleotide to flavin mononucleotide.^26^ We could not detect folate receptor 1 (FOLR1) in the mouse brain; however, it is known that this GPI-AP expresses on the choroid plexus epithelium being involved in the transcytosis of 5-methyl tetrahydrofolate (5MTHF) from blood to cerebrospinal fluid (CSF) and decreased expression of *FOLR1* causes cerebral folate deficiency.^27^ Low concentration of 5MTHF in CSF is often found in IGD cases,^28^ suggesting that *FOLR1* is one of the genes responsible for psychomotor retardation, cerebellar ataxia, and seizures in IGDs.

Despite the effectiveness of AAV-based gene therapy in *Piga*^+/−^ mice, treated mice developed HCC 1 year after treatment. AAV is regarded as nonpathogenic and is considered to be a promising vector for gene delivery. However, recent reports have questioned the safety of AAV. A subset of studies shows that in AAV-treated mice, AAV preferentially integrates into the *Rian* locus, resulting in the overexpression of proximal microRNAs and *Rtl1*, which can lead to carcinogenesis.^10^ The induction of carcinogenesis depends on the AAV dose, enhancer/promoter selection, and the timing of gene delivery. Chicken β-actin (CBA) or liver-specific thyroxine-binding globulin (TBG) promoters plus the CMV enhancer have been hypothesized to promote increased transcription (transactivation) of genes proximal to *Rian* that drive the formation of HCC. The *Rian* locus is highly expressed in neonates and is therefore susceptible to AAV integration. However, the overexpression of microRNAs did not occur when AAV was driven by promoters other than CBA or TBG, indicating that vector-encoded *cis*-regulatory sequences were responsible.^10^ In humans, the upregulation of delta-like homolog 1-deiodinase type 3, the orthologous locus to the mouse *Rian* locus, has been associated with poor survival in patients with hepatic carcinoma.^29^ *RTL1* overexpression activates the Wnt pathway by increasing the levels of DOCK4 and MACF1, both of which enhance the release of β-catenin from the destruction complex and increase the stability of β-catenin in melanoma cells.^30^ Fortunately, no human cases treated with an AAV transgene driven by CBA have developed liver cancer.^31^ It is not known whether the AAV integration preference is different between humans and mice. Unexpectedly, the expression of endogenous *Piga* was increased in the tumor but not the vector-derived *hPIGA* (Figure 8C). This is probably not the cause of carcinogenesis but the result from the Wnt signal activation caused by *Rtl1* overexpression. Consistent with this, the expression of *Pigo,* another GPI biosynthesis gene, or *Glypican3*, the highly expressed GPI-AP in liver cancer, were not drastically increased in the tumor (Figure S8).

Zolgensma (onasemnogene abeparvovec), AAV9-*CAG-SMN1*, has been approved for the treatment of spinal muscular atrophy (SMA) in various countries, including Japan. Most of the treated patients developed liver dysfunction, thrombocytopenia, and thrombotic microangiopathy after AAV administration, and the severity of these adverse effects was correlated with the dose of AAV and its expression level.^32^^,^^33^^,^^34^ This is known to be caused by the immunological reaction to AAV. However, there is no evidence of AAV integration and subsequent HCC in these patients, with the long-term follow-up data suggesting the safety and tolerability of AAV9. Zolgensma treatment in SMA patients is followed up to 7.5 years postdosing. Nonetheless, there are many parameters, such as administration routes, amount of virus, timing of administration, and the promoters, to be considered for the use of AAV to select the safest and most effective method for the gene therapy of IGD.

One limitation of our work is that this mouse model is not an accurate disease model. We could not establish the knockin mouse bearing the same mutation of the affected individual. Individuals with *PIGA* deficiency are always partial deficiencies because complete deficiency is lethal. In these individuals, *PIGA* expression is decreased not only in the CNS but also in whole bodies and expressions of various GPI-APs are decreased to various degrees. AAV-based gene therapy after birth could not completely rescue the mouse phenotypes because *Cre* recombinase driven by *Nestin* promoter completely depletes the *Piga* gene as early as approximately E10 in the CNS, and the defect in brain development due to loss of various GPI-APs at this stage was not reversible. *In utero* administration of *AAVPIGA* would be required to overcome the defects in embryogenesis. As for the gene therapy for partial *PIGA* deficiency, we believe that most of the symptoms are reversible based on the fact that the *PIGO*-deficient mouse model bearing the same mutation of the affected individual was successfully treated with AAV-based gene therapy.^2^

## Materials and methods

### Generation and genotyping of mice

*Piga*-floxed mice were generated in our laboratory.^4^ CNS-specific Cre expressing transgenic mice, B6.Cg-Tg(*Nestin-Cre*) RBRC02412, were provided by the RIKEN BioResource Research Center through the National BioResource Project of the Ministry of Education, Culture, Sports, Science, and Technology, Japan. Homozygous *Piga*-floxed female mice were crossed with *Nestin-Cre* mice. *Piga* is X-linked; therefore, male mice with the Cre transgene were CNS-specific *Piga* KO mice, whereas female mice with the Cre transgene were mosaic for *Piga* expression because of X-inactivation. CNS-specific *Piga* KO mice were sacrificed at the indicated age and whole brains, hearts, and skeletal muscles were taken out. gDNA was isolated after homogenization. Primers for wild-type and *Piga*-floxed alleles were primer 1, 5′-ACCTCCAAAGACTGAGCTGTTG-3′, and primer 2, 5′-CCTGCCTTAGTCTTCCCAGTAC-3′ (fragment sizes 420 and 250 bp, respectively); primers for the targeted allele were primer 1 and primer 3, 5′-TGTGGGTTTCAGTTCATTTCAGA-3′ (fragment size 550 bp) (Figures 1A and 1B); those for the Cre transgene were primer 4, 5′-AGGTTCGTTCACTCATGGA-3′, and primer 5, 5′-TCGACCAGTTTAGTTACCC-3′ (fragment size 235 bp). Mice were maintained in a specific pathogen-free animal facility at the Research Institute for Microbial Diseases, Osaka University, Japan.

### Animals

Mice were maintained under a 12-h light/12-h dark cycle in a temperature-controlled environment, with food and water provided *ad libitum*. All of the animal procedures were approved by the Animal Care and Use Committee of the Research Institute for Microbial Diseases, Osaka University, and were carried out in accordance with the approved guidelines. *Nestin-Cre* transgenic mice were maintained by mating with wild-type C57BL/6 mice and *Piga*-floxed mice were maintained by mating female homozygous *Piga*-floxed mice with hemizygous *Piga*-floxed male mice.

### Histological analysis of the mouse brain and liver

Mice were anesthetized and fixed by cardiac perfusion with 4% paraformaldehyde in 0.1 mol/L phosphate buffer (pH 7.2). Brains were removed from the mice and further immersed in the same fixative overnight at 4°C. Samples processed for paraffin embedding were cut into 5-μm sections with a semimotorized rotary microtome (RM2245; Leica, Nussloch, Germany) and placed on silane-coated glass slides. Samples for cryosections were embedded in optimal cutting temperature compound (Sakura Finetek, Tokyo, Japan) after cryoprotection in 10% and 20% sucrose in 0.1 mol/L phosphate buffer (pH 7.2) and sectioned at 10 μm with a cryostat (CM3050; Leica). The sections were placed on silane-coated glass slides and stored at −80°C until used. Meyer’s H&E staining was performed on paraffin-embedded sections. For MBP immunohistochemistry, deparaffinized sections were stained with rat anti-MBP IgG (no. MCA409S) overnight at 4°C, further incubated with biotinylated goat anti-rat IgG for 1 h, and finally with peroxidase-conjugated streptavidin (Vector Laboratories, Newark, CA) for 1 h at room temperature. Staining for peroxidase was performed using 0.0125% 3,3′-diaminobenzidine tetrahydrochloride and 0.002% H_2_O_2_ in 0.05 mol/L Tris-HCl buffer (pH 7.6) for 10 min. For HA immunohistochemistry, cryosections were incubated with rabbit anti-HA IgG (Cell Signaling Technology, Danvers, MA) and further incubated with fluorescein isothiocyanate (FITC)-conjugated donkey anti-rabbit IgG. Samples were analyzed using a BZ-X800 microscope (Keyence, Osaka, Japan).

### Electron microscopy

Mice were fixed by cardiac perfusion with 2% paraformaldehyde and 2% glutaraldehyde in 0.1 M phosphate buffer (pH 7.2). Brains were removed and 1-mm-thick brain slices were postfixed with 2% paraformaldehyde and 2% glutaraldehyde in 0.1 M phosphate buffer (pH 7.4) overnight followed by postfixation with 1% OsO_4_, dehydration with a graded ethanol series and embedding in Epon812 (Oken Shoji, Tokyo, Japan). Ultrathin sections were cut with an ultramicrotome UC6 (Leica Microsystems), stained with uranyl acetate and lead citrate, and examined with a transmission EM HT7700 microscope (Hitachi, Tokyo, Japan).

### Western blotting

After perfusion with saline, mouse brains were homogenized and solubilized in 60 mM *n*-octyl-β-d-glucoside-containing lysis buffer, followed by centrifugation to remove debris. After bicinchoninic acid assay measurement of protein content, lysates were processed by SDS-PAGE followed by western blotting. The primary antibodies used were rat anti-MBP (MCA409S, Bio-Rad, Hercules, CA) and mouse anti-glyceraldehyde 3-phosphate dehydrogenase (GAPDH) (AM4300, Thermo Fisher Scientific, Waltham, MA). Secondary antibodies used were horseradish peroxidase–conjugated anti-rabbit, anti-rat, or anti-mouse IgG.

### Generation of AAV

pscAAV-*CAG-GFP* was purchased from Addgene (no. 83279). pscAAV-*CAG-2HA-PIGA* was generated from pscAAV-*CAG-GFP* by the replacement of an AgeI/NotI fragment containing GFP with PCR amplified *2HA-human PIGA*. AAV was packaged by the lipofection of pscAAV-*CAG-2HA-hPIGA* with PHPeB capsid^35^ and pAd5 helper plasmid (Addgene) into AAVpro 293T cells (Takara Bio, Kusatsu, Japan), which were purified by polyethylene glycol precipitation followed by iodixanol gradient ultracentrifugation.^36^ Viral titer was determined by qPCR using Taq-Man technology (Thermo Fisher Scientific).

### Intravenous AAV injection into newborn *Piga*-deficient mice

Newborn (P1–P2) *Nestin-Cre*/*Piga*-floxed mice were subjected to intravenous AAV injection as described previously.^37^ Before the procedure, pups were anesthetized by placing on ice for 1 min. They were subsequently injected via the temporal vein using a 30G insulin syringe with needle (Lo-Dose Insulin Syringe with needle, 30G, 1/2 mL, Becton Dickinson, Franklin Lakes, NJ). Pups were then allowed 2–3 min to rewarm and recover and were then returned to their cage.

### MRI

We conducted *in vivo* and *ex vivo* MRI of mice using an 11.7-T vertical bore scanner (AVANCE II 500WB; Bruker BioSpin, Ettlingen, Germany). *In vivo* T_2_ weighted brain MRI images of *Piga*^*+/−*^ mice at 21 days of age and their wild-type littermates was compared (Figure 3A). Mouse anesthesia was initially induced with 2% isoflurane and maintained with 1.6% isoflurane during MRI. Body temperatures of mice were maintained at 37°C with circulating warm water. *In vivo* T_2_ weighted images were obtained by the rapid acquisition with relaxation enhancement technique.^38^ The acquisition parameters were field of view = 15 × 15 mm, matrix size = 256 × 256, in-plane resolution = 59 μm, slice thickness = 300 μm, repetition time = 5000 ms, echo time = 39.5 ms, number of averages = 16, and acquisition time = 21 min.

*Ex vivo* diffusion weighted MRI of mice was performed to compare the brain regions and brain lengths. AAV-treated male *Piga*^*−/*^ mice and their wild-type littermates were imaged at 17 days old. *Ex vivo* images of AAV-treated female *Piga*^*+/−*^ mice and their wild-type littermates were obtained at 19 days old (Figure S2). The acquisition parameters for *ex vivo* diffusion weighted MRI were field of view = 15 × 15 mm, matrix size = 256 × 256, in-plane resolution = 59 μm, slice thickness = 300 μm, repetition time = 5,000 ms, echo time = 19.5 ms, b-value = 3,000 s/mm^2^, number of averages = 6, and acquisition time = 2 h 8 min.

*Ex vivo* T_2_ weighted imaging of AAV-treated Piga^+/−^ mice and their wild-type littermates at 19 and 54 days old was performed (Figure S3). The acquisition parameters for *ex vivo* T_2_ weighted imaging were field of view = 15 × 15 mm, matrix size = 256 × 256, in-plane resolution = 59 μm, slice thickness = 300 μm, repetition time = 5,000 ms, echo time = 50 ms, number of averages = 40, and acquisition time = 53 min.

### Video EEG recordings and analysis

Adult mice at 4 months of age or older were used for EEG recordings as previously described.^2^ In brief, mice were anesthetized with isoflurane and implanted with EEG electrodes (no. 8201: 2 EEG/1 EMG Mouse Headmount, Pinnacle Technology, Parsippany, NJ) according to the manufacturer’s instructions. The electrodes were attached to thin cables linked to a computer running software that allowed the visualization of EEG activity with simultaneous video recording (Vital Recorder, Kissei Comtec, Nagano, Japan). Video EEGs were recorded overnight, and the files were reviewed for background activity, epileptic discharge, and seizure activity (SleepSign, Kissei Comtec, Matsumoto, Japan). In addition, low-dose pentylenetetrazole (20 mg/kg) was administered intraperitoneally, and pentylenetetrazole-induced seizure susceptibility was evaluated using the modified Racine scale.^39^ Frequency and amplitude in 8 h of EEG data (from 8:00 to 4:00) were calculated by fast Fourier transform power spectral analysis.^40^ Data were analyzed in 10-s epochs using SleepSign software. The EEG signal was separated into five regions per epoch. Each region was fast Fourier transform calculated using 256 datum points (2 s) before the 5 spectra were averaged. The spectrum had a resolution of 0.5 Hz.

### Animal behavioral analysis

Muscle weakness and coordination deficit were measured by the four-limb hanging test. The latency to fall was recorded with a 3-min cutoff time. The performance of AAV-treated female variants was compared with that of wild-type controls. Working memory and exploratory activity were measured using a Y-maze apparatus (arm length: 40 cm, arm bottom width: 3 cm, arm upper width: 13 cm, height of wall: 15 cm; BrainScience Idea, Osaka, Japan). Each mouse was put in the Y-maze, one arm of which was blocked, for 5 min. One hour later, each mouse was placed in the bottom area of the Y-maze with both arms open. The number of entries into the arms were recorded for 5 min. Working memory and activity were calculated as the number of correct alterations/number of total new arm entries, as previously described.^41^

### Proteomic analysis of the mouse brain

Six-day-old brains of Nestin-Cre/*Piga*-floxed male mice, AAV-treated mice, and wild-type mice were homogenized and lysed in 500 μL of lysis buffer (10 mM Tris-HCl, pH 7.4, 150 mM NaCl, 5 mM EDTA, protease inhibitor cocktail) containing 2% Triton X-114 (Nakalai Tesque, Kyoto, Japan) for 30 min on ice. After centrifugation at 21,900 × *g* at 4°C for 15 min, the supernatant was incubated for 10 min at 37°C and aqueous and detergent phase separation was performed by centrifugation at 5,600 × *g* at 37°C for 7 min. After recovering the aqueous phase, 350 μL of lysis buffer was added to the detergent phase and further incubated with phosphatidylinositol-specific phospholipase (1 U/mL) at 16°C for 2 h. Phase separation was performed and the aqueous phase was combined with the previously recovered aqueous phase. Four volumes of acetone were added to the combined aqueous and detergent phases, and protein precipitation was performed at −80°C for 1 h. Protein precipitate was pelleted by centrifugation at 13,400 × *g* for 30 min at 4°C and then air dried. The air-dried pellet was dissolved in 20 μL 0.1% RapiGest (Waters, Milford, MA) and reduced with 10 mM DTT, followed by alkylation with 55 mM iodoacetamide, digestion with trypsin, and purification with a C18 tip (AMR, Tokyo, Japan). The purified peptides were subjected to nanocapillary reversed-phase liquid chromatography with tandem mass spectrometry (LC-MS/MS) analysis using a C18 column, Nikkyo NTCC-360 (75 μm × 150 mm, 3.0 μm, Nikkyo Technos, Tokyo, Japan) in a nanoLC system (Bruker Daltonics, Billerica, MA) connected to a timsTOF Pro mass spectrometer (Bruker Daltonics) and a modified nano-electrospray ionization source (CaptiveSpray; Bruker Daltonics). The mobile phase consisted of water containing 0.1% formic acid (solvent A) and acetonitrile containing 0.1% formic acid (solvent B). Linear gradient elution was carried out from 2% to 35% solvent B for 22 min at a flow rate of 400 nL/min. The ion spray voltage was set at 1.6 kV in the positive ion mode. Ions were collected in the trapped ion mobility spectrometry device over 100 ms and MS and MS/MS data were collected over an *m/z* range of 100–2,000. During the collection of MS/MS data, the trapped ion mobility spectrometry cycle was adjusted to 0.53 s and included 1 MS plus 4 parallel accumulation serial fragmentation-MS/MS scans, each containing on average 12 MS/MS spectra (>100 Hz).^42^^,^^43^ Nitrogen gas was used as collision gas. The resulting data were processed using DataAnalysis version 5.2 (Bruker Daltonics), and proteins were identified using MASCOT version 2.7.0 (Matrix Science, London, UK) against the SwissProt database. Quantitative values were calculated with Scaffold 5 (Proteome Software, Portland, OR) for MS/MS-based proteomic studies.^44^ Values were calculated from the sum of all of the spectra from a specific protein referred to the total spectrum counts. Each sample was normalized with the total spectrum counts.

### Measurement of virus-derived *PIGA* expression in various tissues and endogenous *Rtl1* in liver cancers

At 1 year of age, AAV-treated mice and wild-type littermates (n = 3) were euthanized and tissues were dissected. Total RNA was isolated with an RNAeasy kit (Qiagen, Hilden, Germany) after homogenization and gDNA was removed. Total RNA was reverse transcribed using a Superscript VILO kit (Thermo Fisher Scientific) and qPCR was performed with the cDNA using SYBR Green PCR mater mix (Thermo Fisher Scientific) and primers on the StepOnePlus Real-Time PCR System (Thermo Fisher Scientific) with the comparative Ct method. (The primers used for qPCR were, for mPiga: 5′- GTGAAGTCGGGGACATTGCC and 5′- GCAAACATGTAGCCCGTCAC; for hPIGA: 5′- GGGACATTGCCAGCTCCAGA and 5′- TCTGTCCAGTCGTTTGTCCATTGG; for mPigo: 5′-GCAGTAACTTTGCCAGCCATGC and 5′- TAGAGGTGTTCCAAGATGCCG; for mRtl1: 5′- TACTGCTCTTGGTGAGAGTGGACCC and 5′- GGAGCCACTTCATGCCTAAGACGA; for mGpc3: 5′- CAGCCCGGACTCAAATGGG and 5′- AGCCGTGCTGTTAGTTGGTATTTTTC; for endogenous control: TATA box binding protein; and for mTbp: 5′-TATGACCCCTATCACTCCTG and 5′-TTCTTCACTCTTGGT.).

As for comparison of AAV-derived *hPIGA* expression level with endogenous *mPiga* expression level, copy number standard curves were generated by the dilution of *hPIGA* and *mPiga* expression plasmids. The copy numbers of *hPIGA* and *mPiga* mRNA of AAV-treated mice were analyzed and normalized with *Tbp* expression.

As for the time course analysis, 3 pregnant mice were purchased from CLEA Japan, and 16 pups were intravenously injected with 10^11^ vg/mouse of AAV *PHPeB hPIGA.* The pups were divided into 4 groups and 4 mice each were sacrificed at days 4, 10, 15, and 25 after injection for qPCR analysis of *hPIGA* expression with the comparative Ct method mentioned above.

## Data and code availability

All of the data are available from the corresponding author on reasonable request.

## Supplemental information

Document S1. Figures S1–S8 and Table S1

Document S2. Article plus supplemental information
