# Supplementary material for: AAV-based gene therapy ameliorated CNS-specific GPI defect in mouse models
Source: Mol Ther Methods Clin Dev. 2023 Dec 14;32(1):101176. doi: 10.1016/j.omtm.2023.101176 (PMC10788267; doi:10.1016/j.omtm.2023.101176)
Supplement: Document S1. Figures S1–S8 and Table S1 [file mmc1.pdf]

OMTM, Volume *32*

Supplemental information

AAV-based gene therapy ameliorated

CNS-specific GPI defect in mouse models

Yoshiko Murakami, Saori Umeshita, Kae Imanishi, Yoshichika Yoshioka, Akinori Ninomiya, Takehiko Sunabori, Shibi Likhite, Masato Koike, Kathrin C. Meyer, and Taroh Kinoshita

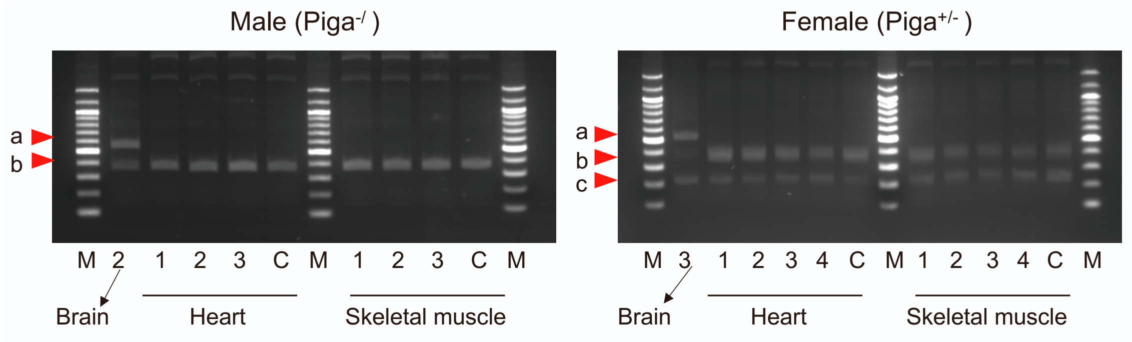

Figure S1. Genotypes of heart and skeletal muscle from three *Piga*$^{-/}$ (day 1) and four *Piga*$^{+/-}$ mice (1, 2: day4, 3, 4: day1) Brain (cerebrum) sample (day1) is the positive control for exon6 deletion; C is the sample from the *Pigafloxed* mouse (day1) for the negative control. a, *Piga* exon6 deleted; b, *Piga floxed*; c, Wild type

A.

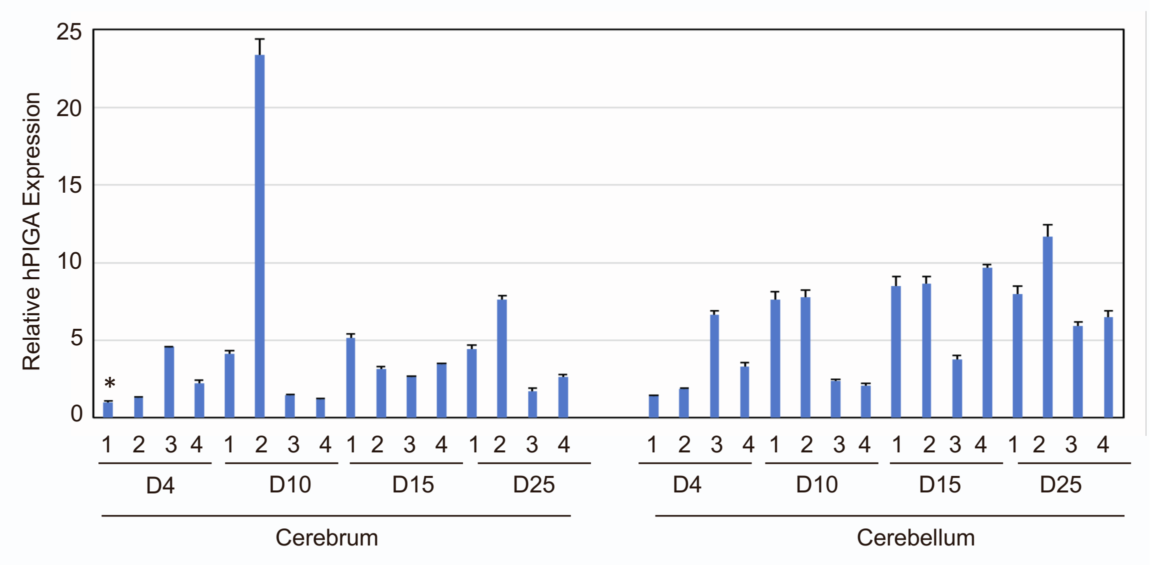

B.

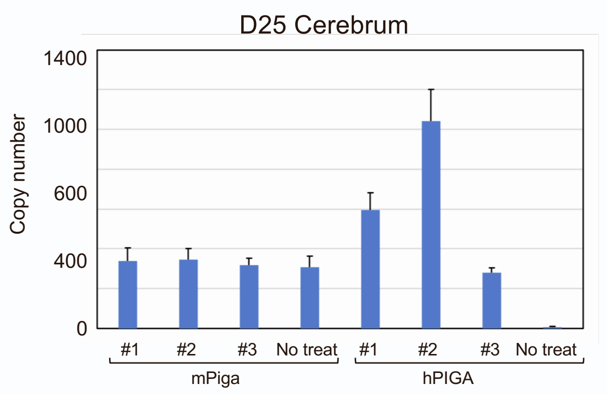

Figure S2. Expression of AAV-derived hPIGA at each time point after administration of AAV A. Relative expression of *hPIGA* in the cerebrum and the cerebellum of AAV-treated mice at suggested time points. 1-4 indicates individual mouse in each group. Expression of cerebrum D4 #1(*) was set to 1.

B. Comparison of mRNA copy number between AAV-derived *hPIGA* and endogenous *mPiga* in D25 AAV-treated Cerebrum.

.

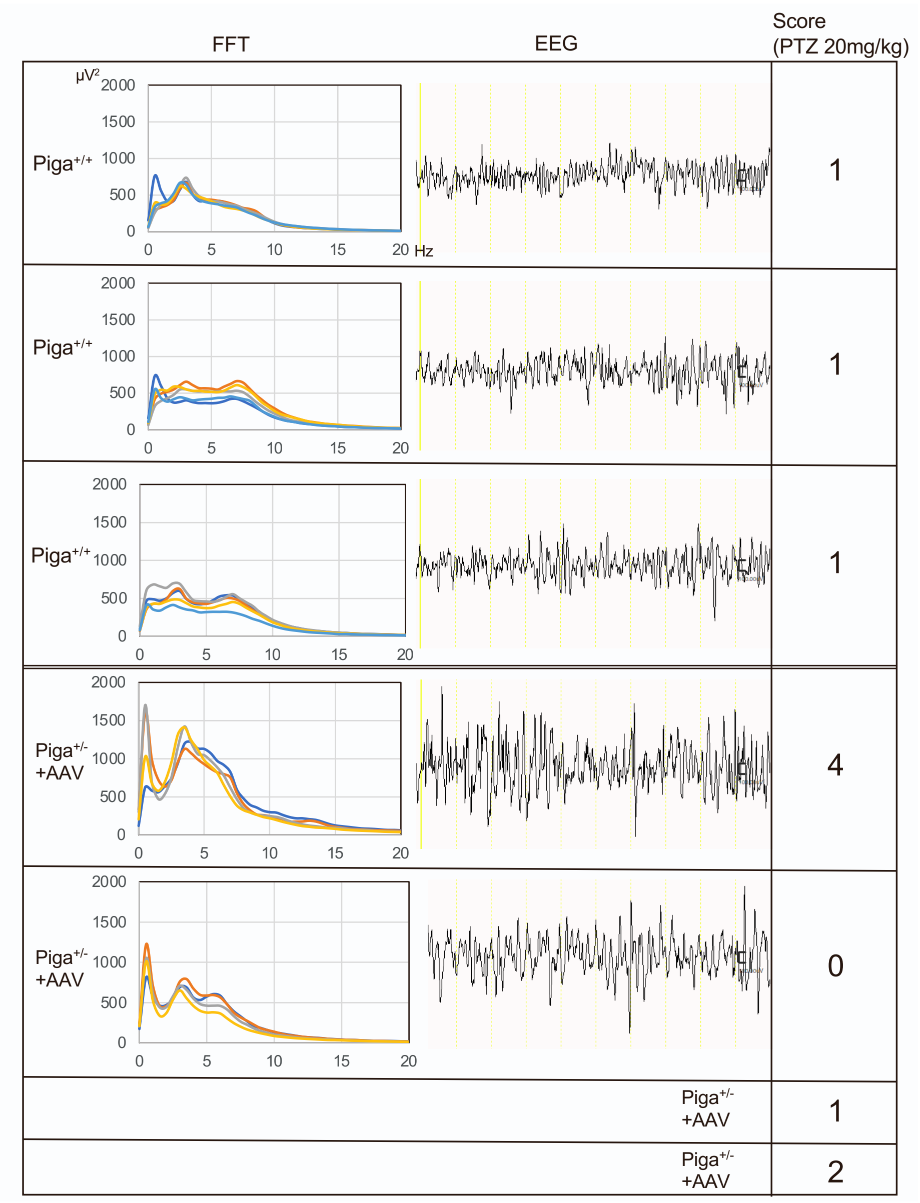

Figure S3. Fast Fourier transform (FFT) power spectral analysis of EEG background activity Power spectrum of average FFT of 8-hour recordings in dark phase, showing data for two AAV-treated *Piga*$^{+/-}$ mice and three wild-type littermates with representative images of background EEG activity. Colored lines show the FFT spectrum of each 2-hour EEG recording within 8 hours in total. Seizure susceptibility induced by a single injection of low-dose pentylenetetrazole (20 mg/kg) is shown using the previously described pentylenetetrazole scale (5) (Table S1) (wild-type n=3, AAV-treated *Piga*$^{+/-}$ mice, n=4).

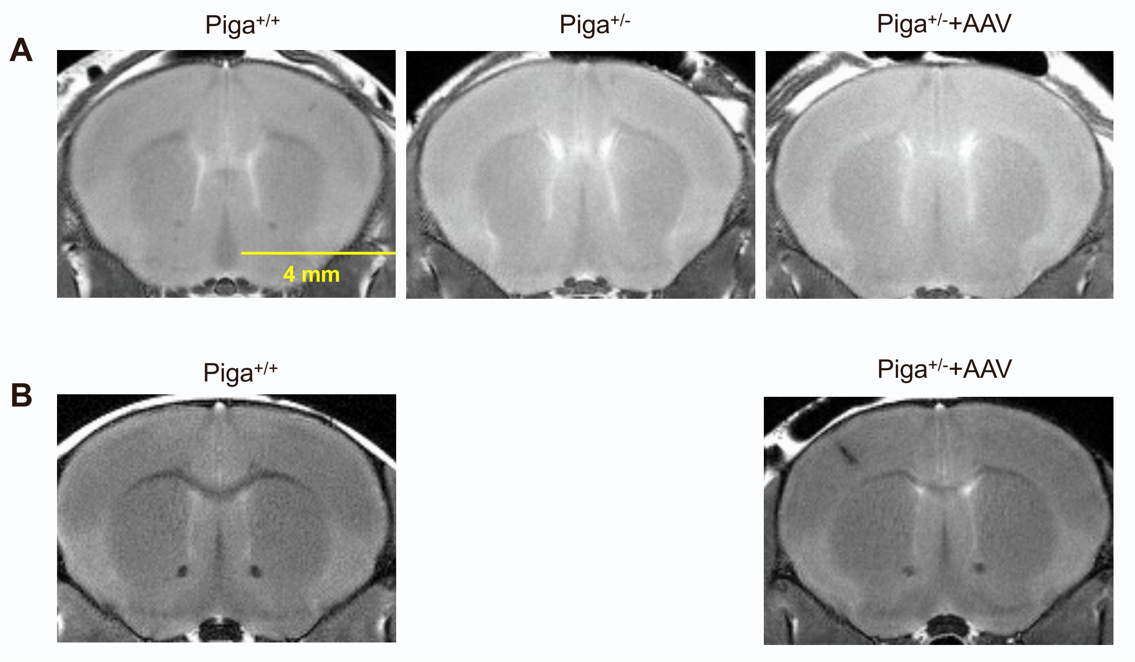

Figure S4. ***In vivo*** **T_2 weighted brain MRI of AAV-treated** ***Piga*** **$^{+/-}$ mice compared with wild-type littermates A.** Brain MRI of an AAV-treated *Piga*$^{+/-}$ mouse at 19 days old compared with its wild-type and non-treated littermates. **B.** Brain MRI of an AAV-treated *Piga*$^{+/-}$ mouse at 54 days old compared with its wild-type littermate.

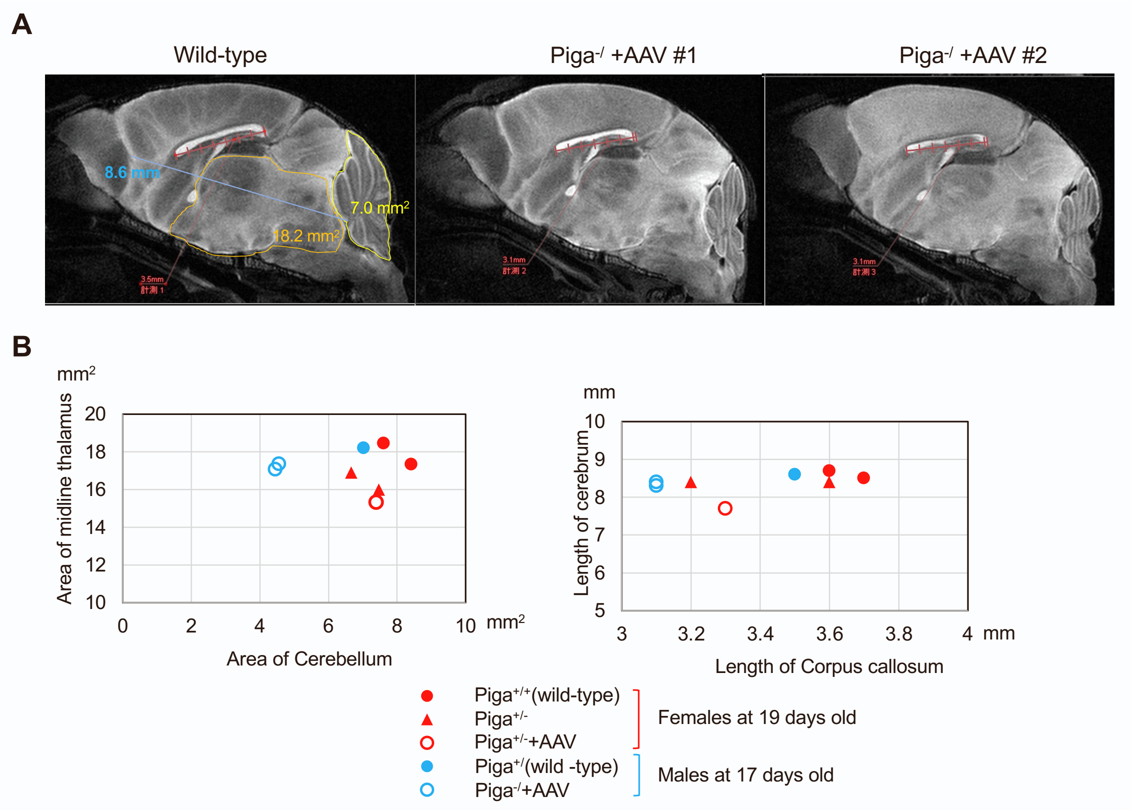

Figure S5. MRI measurement of brain regions A. Sagittal brain sections of AAV-treated *Piga*$^{-/}$ mice compared with a wild-type littermate at 17 days old. **B.** Measurement of various brain regions of AAV-treated *Piga*$^{-/}$ and *Piga*$^{+/-}$ mice compared with wild-type or non-treated littermates. AAV-treated male *Piga*$^{-/}$ mice and their wild-type littermates at 17 days old and AAV-treated female *Piga*$^{+/-}$ mice and their littermates at 19 days old.

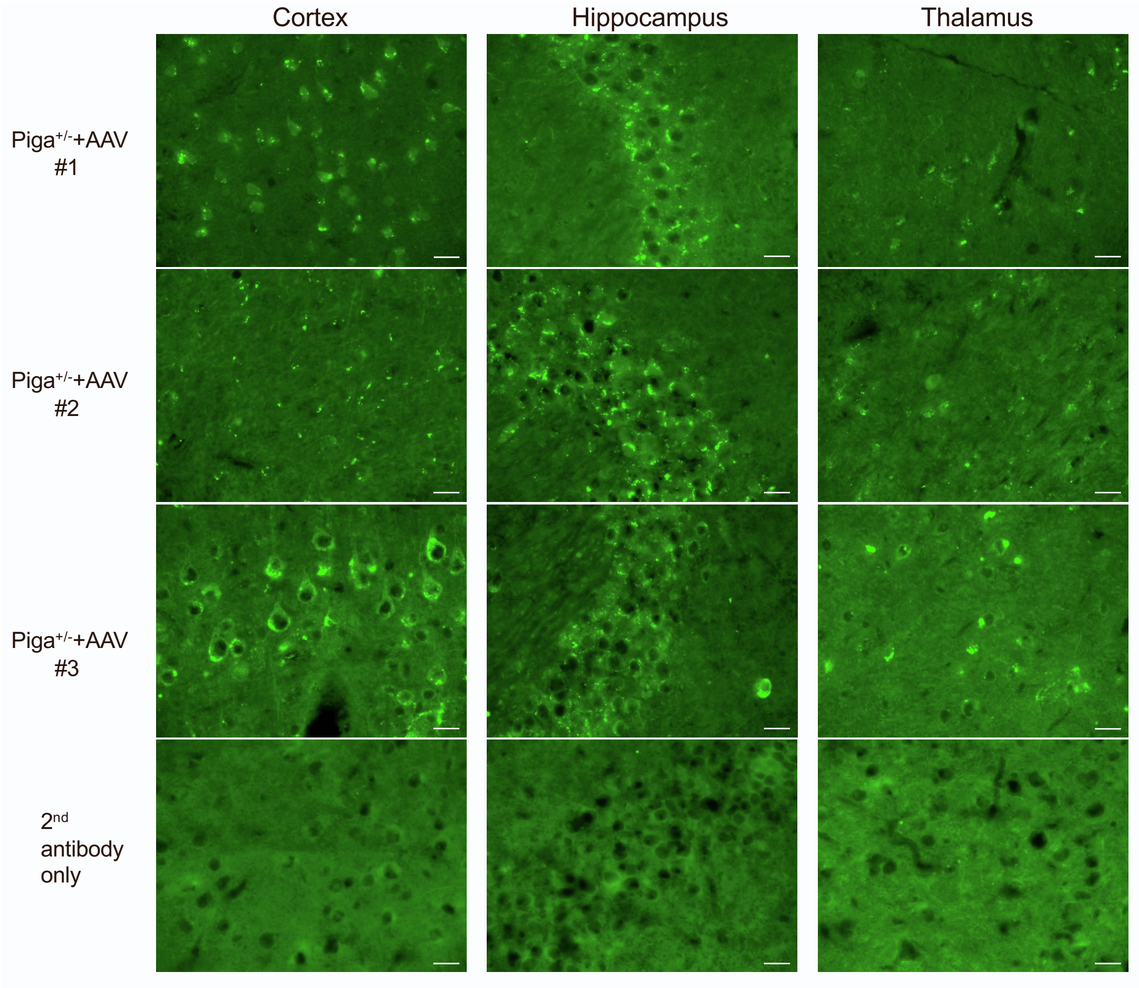

Figure S6. Anti-HA staining (for HA-hPIGA) in various regions of the brain of AAV-treated *Piga*$^{+/-}$ mice at 1 year old Samples were stained with an anti- HA antibody, followed by FITC-conjugated anti-rabbit IgG. Images are 60×; scale bars, 20 μm.

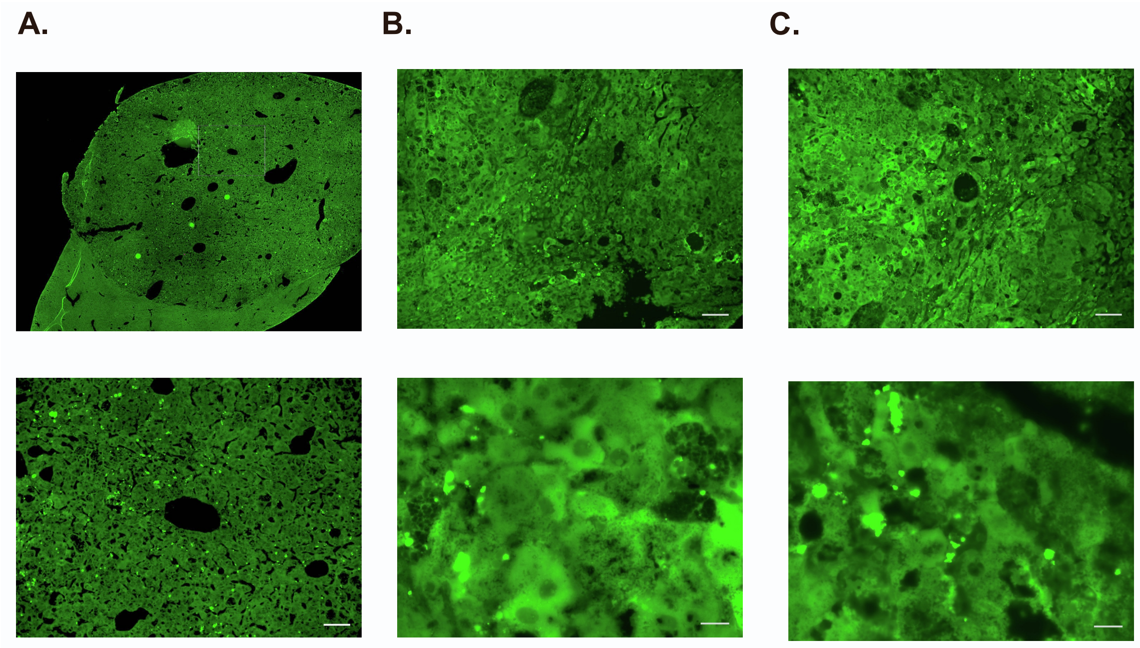

Figure S7. Anti-HA staining of the liver tumor A. A liver tumor from an AAV-treated *Piga*$^{+/-}$ mouse (#3); upper, a navigation image of whole tumor; lower, an 11.1× image. **B.** upper, an 11.1× image of the same liver tumor as A but the different part; lower, a 60× image. C. Control staining with the secondary antibody only of B; upper, 11.1× image; lower, 60× image, showing green dots were also appeared in the control staining. No specific staining of HA-PIGA was detected in the tumor. Scale bars in 11.1× images, 100 µm; in 60× images, 20 µm.

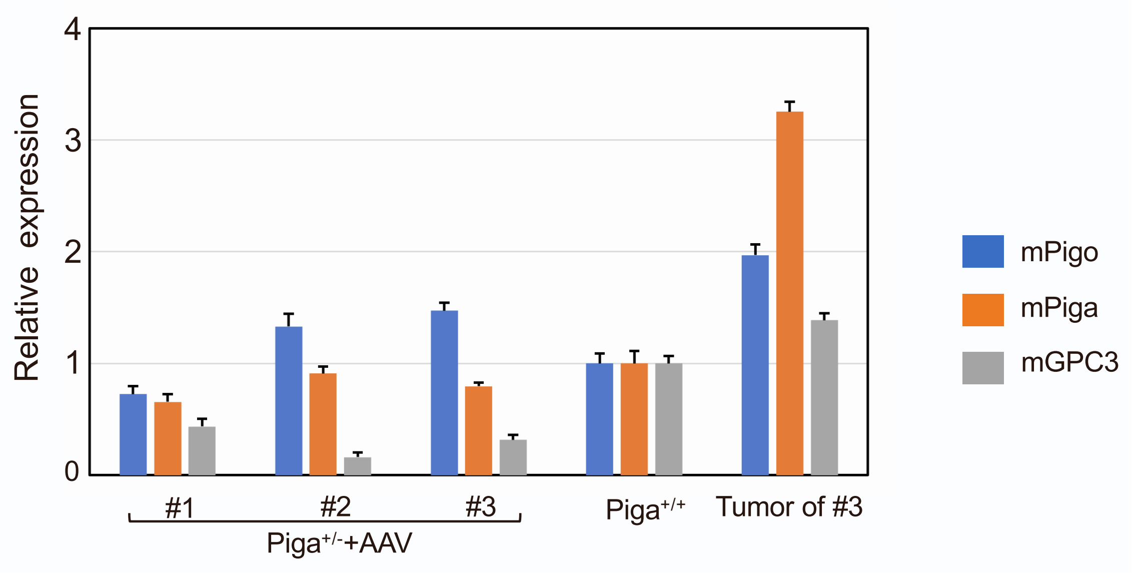

Figure S8. Relative expression of endogenous *Piga, Pigo and Glypican3* in the liver of AAV-treated aged *Piga*$^{+/-}$ mice, which developed liver tumors Relative expressions of *Piga, Pigo and Glypican3* in normal parts of the liver and in tumors of AAV-treated mice compared to those of the wild-type mice.

Table S1

New pentylenetetrazole scale referring to the modified Racine scale

Score	Behavioral stage	EEG findings
0	Normal	Wake rhythm
1	Normal	Spike-wave discharges
2	Whisker trembling, sudden behavioral arrest, facial jerking	EEG slowing, increased amptitude, intermittent spike-wave discharges
3	Neck jerks	Sharp spikes, followed by spike-wave discharges
4	Clonic seizures (sitting)	High frequency, small amplitude rhythmic waves
5	Clonic, tonic-clonic seizure (lying on belly)	High-amplitude polyspikes, spike-wave discharges
6	Clonic, tonic-clonic seizure (lying on side) & wild jumping	High-amplitude polyspikes, spike-wave discharges
7	Tonic extension, possibly leading to respiratory arrest and death	EEG trace appears almost flat
